# Prognostic significance of nestin expression in patients with resected non-small cell lung cancer treated with platinum-based adjuvant chemotherapy; relationship between nestin expression and epithelial to mesenchymal transition related markers

**DOI:** 10.1371/journal.pone.0173886

**Published:** 2017-03-30

**Authors:** Shinichiro Ryuge, Yuichi Sato, Ryo Nagashio, Yasuhiro Hiyoshi, Ken Katono, Satoshi Igawa, Hiroyasu Nakashima, Kazu Shiomi, Masaaki Ichinoe, Yoshiki Murakumo, Makoto Saegusa, Yukitoshi Satoh, Noriyuki Masuda

**Affiliations:** 1 Department of Respiratory Medicine, School of Medicine, Kitasato University, Kanagawa, Japan; 2 Department of Molecular Diagnostics, School of Allied Health Sciences, Kitasato University, Kanagawa, Japan; 3 Department of Respiratory Medicine, Sagamihara Kyodo Hospital, Kanagawa, Japan; 4 Department of Thoracic Surgery, School of Medicine, Kitasato University, Kanagawa, Japan; 5 Department of Pathology, School of Medicine, Kitasato University, Kanagawa, Japan; University of South Alabama Mitchell Cancer Institute, UNITED STATES

## Abstract

**Introduction:**

Although adjuvant platinum-based chemotherapy (AC) has been shown to improve survival of patients with completely resected stage II and stage IIIA non-small cell lung cancer (NSCLC), its effect is limited. Nestin is a class VI intermediate filament protein expressed in neural stem cells and several cancer cells including NSCLC. In the present study, we aimed to determine its prognostic significance concerning survival in NSCLC patients receiving AC.

**Methods:**

Nestin expression in cancer cells was immunohistochemically studied in 90 patients with completely resected stage II and stage IIIA NSCLC treated with AC and its association with clinicopathologic parameters, including ABCG2, E-cadherin, and vimentin expression, was evaluated. Kaplan-Meier survival analysis and Cox proportional hazards models were used to estimate the effect of nestin expression on survival.

**Results:**

Nestin expression was observed in 28 of the 90 (31.1%) NSCLCs. Clinicopathologically, nestin expression was associated with loss of E-cadherin expression (*P* = 0.006) and vimentin positive expression (*P* < 0.001). In survival analysis, nestin expression was significantly associated with a poorer prognosis (*P* = 0.028). Multivariable analysis confirmed that nestin expression is an independent prognostic indicator in NSCLC patients receiving AC (HR = 2.56; 95% CI, 1.23–5.30, *P* = 0.01).

**Conclusion:**

The present study reveals that nestin expression is a prognostic indicator of a poorer survival probability in NSCLC patients receiving AC, although its prognostic significance still requires confirmation with larger patient populations.

## Introduction

Primary lung cancer is the leading cause of cancer mortality worldwide [1]. While surgical resection is the optimal treatment of early-stage of non-small cell lung cancer (NSCLC), 5-year survival rates for surgically resectable NSCLC are still unsatisfactory, and range from 19% for stage IIIA to 63% for stage IA [2]. Although adjuvant platinum-based chemotherapy (AC) has been recommended to improve survival of patients with completely resected stage II and stage IIIA NSCLC, which shows some improvement of 5-year overall survival (ranges from 4% to 15%) [3, 4], its effect is limited.

Nestin is a class VI intermediate filament protein that is specifically expressed in stem/progenitor cells of the developing central nervous system [5]. Nestin is an extensively studied marker of neural stem cells that is a putative marker of the cancer stem cell (CSC) phenotype, as its expression has been identified in many human malignancies [6]. We previously reported that nestin is expressed in a subset of NSCLC and its expression is related to clinicopathological factors, and that nestin expression is a prognostic indicator of poor survival in patients with resected NSCLC [7]. Regarding drug resistance in cancer cells, it is suggested that cancer stem cells are resistant to chemotherapy through their quiescence, their capacity for DNA repair, and their ATP-binding cassette (ABC) transporter expression [8]. Given that nestin-positive tumor cells have characteristics of CSCs, those nestin-positive tumor cells may be resistant to chemotherapy. To our knowledge, no report has been published concerning the relationships between nestin expression and clinicopathological features and prognosis in resected NSCLC patients receiving AC. Therefore, the aims of the present study were: (1) to immunohistochemically examine nestin expression in tumor cells of NSCLCs, (2) to evaluate the relationships between nestin expression in tumor cells and the clinicopathological parameters, (3) to immunohistochemically examine the role of ABC transporter family, ATP-binding cassette sub-family G member 2 (ABCG2) expression and epithelial to mesenchymal transition (EMT)-related markers such as E-cadherin and vimentin expression in the relationship between nestin expression and chemoresistance, and (4) to estimate the prognostic impact of nestin expression on survival of resected NSCLC patients receiving AC.

## Materials and methods

### Ethics statements

The study was approved by the Ethics Committee of the Kitasato University School of Medicine (B15-74) and followed the Declaration of Helsinki protocol. All patients were approached based on approved ethical guidelines, agreed to participate in this study, and could refuse entry and discontinue participation at any time. All participants proved written consent.

### Patients and tissue specimens

A total of 90 consecutive NSCLC patients with completely resected stage II and stage IIIA treated with AC from January 2003 to September 2012 at the Kitasato University Hospital were included in this retrospective cohort study. Eleven of 90 patients were also included in our previous study [7]. Patients who received preoperative chemotherapy and/or radiotherapy were excluded. The histological diagnosis was defined according to the World Health Organization/International Association for the Study of Lung Cancer (WHO/IASLC) classification of lung and pleural tumors [9]. The pathologic TNM (p-TNM) staging was defined according to the 7th Edition of TNM classification [10]. We reviewed each patient’s record to obtain the clinical and pathologic parameters and analyzed for each case. DFS was estimated as the time from surgery to recurrence or death from the disease. OS was defined as the duration from the date of surgery to the date of death or the end of the follow-up.

### Immunohistochemical staining

Ten-percent formalin-fixed and paraffin-embedded tissues were processed into 3-μm-thick sections. The sections were reacted with 100-times-diluted anti-nestin antibody (clone N1602, IBL; Takasaki, Japan) for 2 hrs at room temperature. The details of the procedure were described previously from our laboratory [7].

For ABCG2, E-cadherin, and Vimentin immunohistochemical staining, for antigen retrieval, the sections were autoclaved in 0.01 mol/L citrate buffer (pH 6.0) with 0.1% Tween 20 at 121°C for 10 min. The sections were reacted with 200-times-diluted anti-ABCG2 antibody (clone BXP-21, Abcam; Cambridge, UK) and anti-Vimentin antibody (clone V9, DAKO; Glostrup, Denmark) for 2 hrs at room temperature, and 200-times-diluted anti-E-cadherin antibody (clone HECD-1, Takara; Kusatsu, Japan) for 1 hr at room temperature.

### Evaluation of immunohistochemical staining

For nestin and ABCG2, cytoplasmic immunostaining in tumor cells was considered to be positive. The stainability of peritumoral vascular endothelial cells in nestin and bronchial epithelial cells in ABCG2 was used as an internal positive control. The staining intensity was categorized into 4 groups by comparing the staining intensity of internal positive control: 0 = negative, 1 (weak) = weaker than positive control, 2 (moderate) = the same as positive control, and 3 (strong) = stronger than positive control. Tumors with a staining score of 2 or 3 were judged as positive. Immunohistochemical staining was semi-quantitatively assessed based on the approximate percentage of positive cells over the total number of tumor cells and was determined according to the criteria described previously as follows: nestin negative: <5%, nestin positive: ≥5% [7]; ABCG2 negative ≤10%, ABCG2 positive: >10% [11].

For E-cadherin and vimentin, cell membrane immunostaining in tumor cells was considered to be positive in E-cadherin, and cytoplasmic immunostaining in tumor cells was considered to be positive in vimentin. According to previous studies [12, 13], evaluation of the cell staining reaction was performed in accordance with the following immunoreactive score (IRS) as follows: IRS = SI (staining intensity) × PP (percentage of positive tumor cells). SI was defined as 0, negative; 1, weak; 2, moderate; and 3, strong. PP was defined as 0, negative; 1, 1–10% positive tumor cells; 2, 11–50% positive tumor cells; 3, 51–80% tumor cells; and 4, >80% positive tumor cells. IRS value ≥ 4 was considered as a positive staining result. Moreover, based on the combined results from expression of E-cadherin and vimentin proposed by Sung et al. [14], we used the following 4 phenotypes of EMT: (1) complete type, characterized by loss of the epithelial phenotype with acquisition of the mesenchymal phenotype; (2) incomplete type 1 (hybrid type), characterized by a tumor showing both epithelial and mesenchymal phenotypes; (3) incomplete type 2 (null type), defined by loss of the epithelial phenotype without acquisition of the mesenchymal phenotype; and (4) wild type, characterized by a tumor with no evidence of EMT. Two investigators (R. S. and S. Y.) separately evaluated all the specimens without clinicopathological information. Variant cases were reviewed and discussed until a consensus was obtained for each of the specimens.

### Statistical analysis

All statistical analyses were performed using SPSS version 23.0 software (SPSS Inc., Chicago, Illinois). In immunohistochemical analysis, inter-rater reliability between two investigators was calculated using the kappa statistic. The kappa coefficient was interpreted according to the following coefficient proposed by Landis and Koch: ≤ 0.20 (slight agreement); 0.21–0.40 (fair); 0.41–0.60 (moderate); 0.61–0.80 (substantial); and 0.80–1.00 (almost perfect) [15]. The relationships between nestin expression and clinicopathological parameters were estimate by Pearson’s χ^2^ test or Fisher’s exact test, as appropriate. DFS and OS of patients was estimated by using the Kaplan-Meier method and compared by the log-rank test. A multivariable analysis was estimated by using the Cox proportional hazards model. If the *P*-value was less than 0.05, differences were considered significant. All reported P-values are two-sided.

## Results

### Patient characteristics

The clinicopathological characteristics of the patients are summarized in Table 1. There were 66 (73.3%) patients with adenocarcinomas (ADs), 18 (20.0%) with squamous cell carcinomas (SCCs), 3 (3.3%) with pleomorphic carcinomas (PCs), 2 (2.2%) with large cell neuroendocrine carcinomas (LCNECs), and 1 (1.1%) with adenosquamous carcinoma. The overall follow-up durations ranged from 5 to 149 months (median, 52 months). No patients died from treatment-related death. A total of 49 patients were alive at the end of the follow-up, 32 patients died of lung cancer, 3 patients died from other causes, and 6 patients were lost to follow-up.

**Table 1:** Characteristics of the patients.

Characteristics	Patients (N = 90)
**Age, y**	
Median age (range)	61 (40–75)
**Gender**	
Male	55 (61.1)
Female	35 (38.9)
**Smoking habit**	
Smoker	35 (38.9)
Never smoker	55 (61.1)
**Histological type**	
AD	66 (73.3)
Non-AD	24 (26.7)
**p-TNM stage**	
Stage II	28 (31.1)
Stage IIIA	62 (68.9)
**Chemotherapeutic regimen**	
CBDCA-based	29 (32.2)
CDDP-based	61 (67.8)
**Number of treatment cycle**	
Median cycle (range)	3 (1–4)
**Vital status**	
Alive	49 (54.4)
Lung cancer-related death	32 (35.6)
Other causes of death	3 (3.3)
Unknown	6 (6.7)

Data are presented as No. (%) unless otherwise indicated.

AD = adenocarcinoma; CBDCA = carboplatin; CDDP = cisplatin;

p-TNM = pathologic TNM.

### Nestin expression in NSCLC

**Fig 1:**
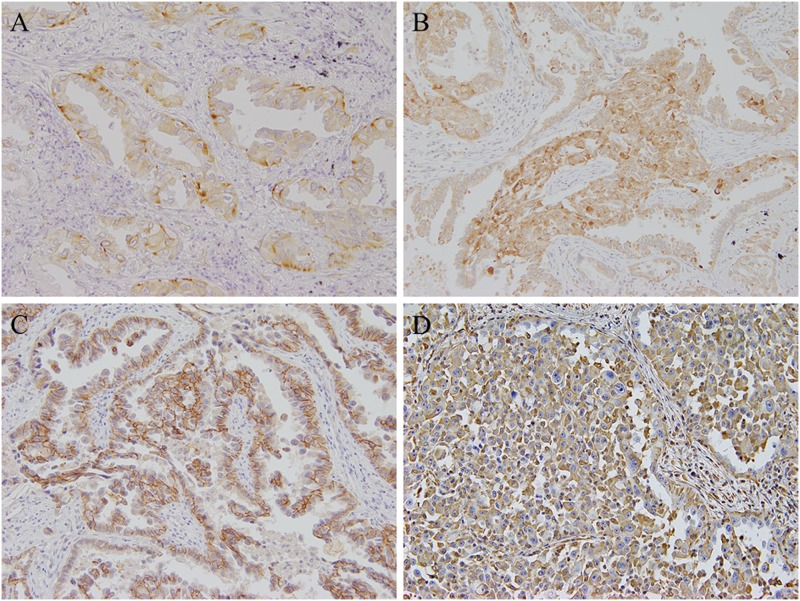
Representative images of immunohistochemical staining of NSCLC samples. (A) Nestin, (B) ABCG2, (C) E-cadherin, and (D) Vimentin show positive expression. (original magnification, ×200).

Cytoplasmic nestin expression in tumor cells was observed in 28 of 90 (31.1%) NSCLCs (Fig 1). These were further divided into 18 of 66 (27.3%) ADs, 6 of 18 (33.3%) SCCs, 3 of 3 (100%) PCs, and 1 of 2 (50%) LCNECs. Nestin expression was also observed in the cytoplasm of vascular endothelial cells and fibroblasts in tumor stroma in each case. Nestin expression was not detected in non-neoplastic bronchial or alveolar epithelial cells. No expression was observed in the negative controls.

### The kappa coefficient for inter-rater reliability between two investigators on nestin, ABCG2, E-cadherin, and vimentin expression

Inter-rater reliability between two investigators on nestin expression was in almost perfect agreement with the kappa coefficient of 0.872. Inter-rater reliabilities between two investigators on ABCG2, E-cadherin, and vimentin expressions were in substantial agreement with the kappa coefficients of 0.712, 0.755, and 0.779, respectively.

### Relationship between nestin expression and clinicopathologic characteristics including ABCG2, E-cadherin, and vimentin expression

**Fig 2:**
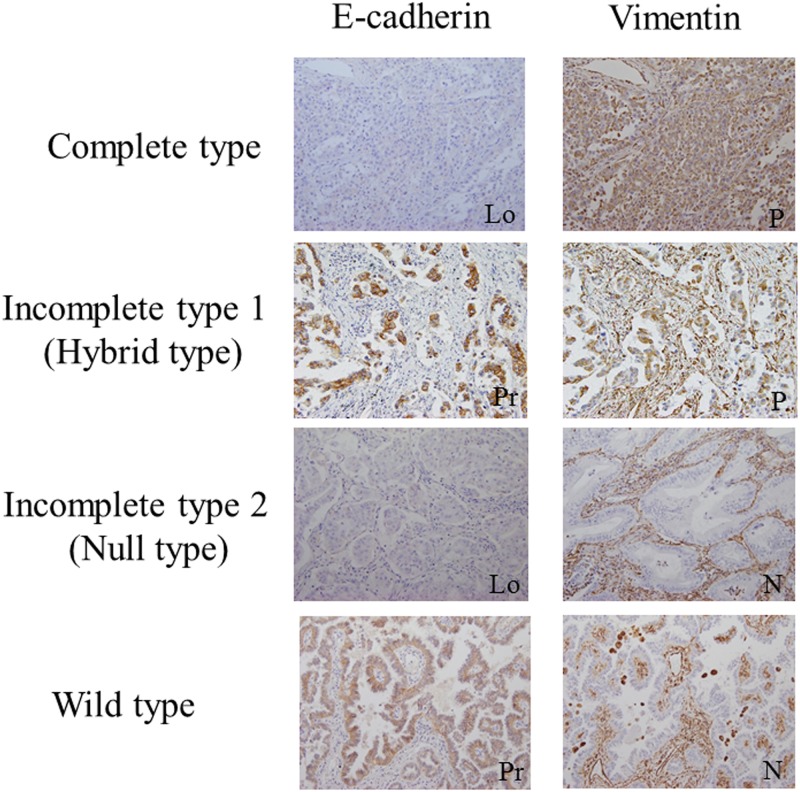
Representative cases of each EMT phenotype according to expression of E-cadherin and vimentin. The cases are divided into the following 4 phenotypes; complete type, incomplete type1 (hybrid type), incomplete type2 (null type), and wild type (Lo, loss; Pr, preserve; P, positive; N, negative. original magnification, ×200).

The relationships between nestin expression and clinicopathologic characteristics are summarized in Table 2. Immunohistochemical analysis showed that nestin expression was not associated with ABCG2 expression (*P* = 0.18). However, nestin expression was significantly associated with loss of E-cadherin expression (*P* = 0.006) and positive expression of vimentin (*P* < 0.001). Nestin expression and EMT phenotype according to expression of E-cadherin and vimentin are showed in Table 2 and Fig 2. Additionally, nestin expression was significantly associated with the complete type in the EMT phenotype (*P* < 0.001). In the present study, 31 patients were analyzed for EGFR mutation status. EGFR common mutation (Del 19 or L858R) was detected in 20 of 31 (64.5%) patients. These were then divided into 4 of the 7 (57.1%) nestin-positive group and 16 of the 24 (66.7%) nestin-negative group, and the status of EGFR mutation was not different between nestin-positive and nestin-negative groups (*P* = 0.67).

**Table 2:** Relationships between nestin expression and clinicopathological parameters.

Clinicopathological Parameters	Nestin Expression		Total	*P*-Value
	Positive (N = 28)	Negative (N = 62)		
**Age, y**				0.24
< 60	9 (24.3)	28 (75.7)	37	
≥ 60	19 (35.8)	34 (64.2)	53	
**Gender**				0.64
Male	18 (32.7)	37 (67.3)	55	
Female	10 (28.6)	25 (71.4)	35	
**Smoking habit**				0.32
Smoker	15 (27.3)	40 (72.7)	55	
Never smoker	13 (37.1)	22 (62.9)	35	
**Histological type**				0.19
AD	18 (27.3)	48 (72.7)	66	
Non-AD	10 (41.7)	14 (58.3)	24	
**p-TNM stage**				0.18
Stage II	6 (21.4)	22 (78.6)	28	
Stage IIIA	22 (35.5)	40 (75.4)	62	
**Chemotherapeutic regimen**				0.053
CBDCA-based	13 (44.8)	16 (55.2)	29	
CDDP-based	15 (24.6)	46 (75.4)	61	
**Number of treatment cycle**				1.00
1, 2	4 (30.8)	9 (69.2)	13	
3, 4	24 (31.2)	53 (68.8)	77	
**Immunohistochemistry**				
**ABCG2**				0.18
Negative	22 (28.2)	56 (71.8)	78	
Positive	6 (50.0)	6 (50.0)	12	
**E-cadherin**				0.006
Loss	19 (46.3)	22 (53.7)	41	
Preserved	9 (18.4)	40 (81.6)	49	
**Vimentin**				< 0.001
Negative	11 (17.2)	53 (82.8)	64	
Positive	17 (65.3)	9 (34.6)	26	
**ENT phenotype**				
Complete type	11 (78.6)	3 (21.4)	14	
Incomplete type 1 (Hybrid type)	6 (50.0)	6 (50.0)	12	
Incomplete type 2 (Null type)	8 (29.6)	19 (70.4)	27	
Wild type	3 (8.1)	34 (91.9)	37	
**ENT phenotype**				< 0.001
Complete type	11 (78.6)	3 (21.4)	14	
Incomplete type, Wild type	17 (22.4)	59 (77.6)	76	

Data are presented as No. (%). EMT = epithelial to mesenchymal transition.

See Table 1 legend for expansion of abbreviations.

### The effect of nestin expression on survival

**Fig 3:**
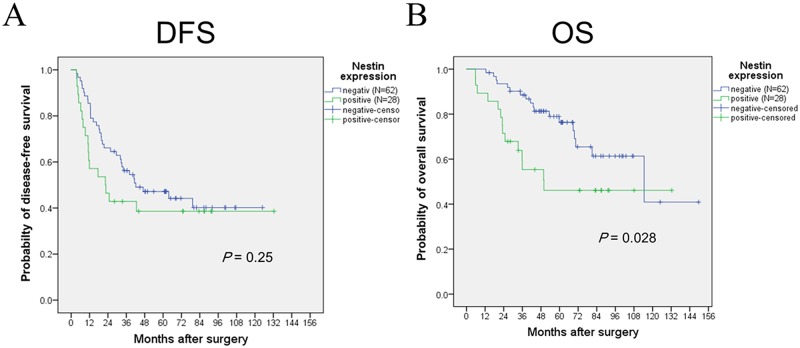
Cumulative survival of patients with NSCLC according to nestin expression estimated by the Kaplan-Meier method. Panel (A) and (B) shows disease-free survival (DFS) and overall survival (OS), respectively. Nestin expression is significantly associated with poorer survival in patients with resected non-small cell lung cancer treated with platinum-based adjuvant chemotherapy, whereas there was no association between DFS and nestin expression.

All the patients were included in the survival analysis. The overall follow-up periods ranged from 5.8 to 149.7 months (median, 52.3 months), the median DFS for all patients was 41.0 months (95% confidence interval (CI), 27.0–54.9). The median DFS was not significantly different, with 22.3 months for patients with nestin-positive tumors and 42.3 months for those with nestin-negative tumors (*P* = 0.256, Fig 3A).

The median OS for all patients was 114.5 months (95% CI, 53.8–175.1). The median OS was 49.9 months for patients with nestin-positive tumors and 114.5 months for those with nestin-negative tumors, and nestin expression was significantly associated with a poorer prognosis (*P* = 0.028, Fig 3B). Moreover, we estimated the prognostic impact of nestin expression on survival in 20 patients with EGFR mutations. There was no difference in OS between nestin- positive and nestin-negative groups (*P* = 0.54).

### Effect of nestin expression on survival with multivariable analysis

Univariable analysis was performed according to Cox proportional hazard regression model to evaluate the effect of nestin expression and other clinicopathologic variables on survival. The results indicated that histological type, p-TNM stage, and nestin expression were significant prognostic factors for survival. Furthermore, nestin expression and other clinicopathologic variables, including histological type and p-TNM stage, were entered into multivariable analysis using the Cox proportional hazard regression model. The results indicated that nestin expression was a significant independent risk factor of poorer survival (HR = 2.56; 95% CI, 1.23–5.30; *P* = 0.011, Table 3).

**Table 3:** Uni- and multivariable analyses of the effect of nestin expression on survival.

Factors	Univariable Analysis			Multivariable Analysis		
	HR	95% CI	*P*-Value	HR	95% CI	*P*-Value
**Nestin expression**						
Positive vs. Negative	2.15	1.09–4.35	0.032	2.56	1.23–5.30	0.011
**Age**						
≥ 60 y vs. < 60 y	1.65	0.77–3.50	0.19	Not included in analysis		
**Gender**						
Male vs. Female	0.68	0.33–1.37	0.28	Not included in analysis		
**Smoking habits**						
Smokers vs. Never Smokers	0.90	0.44–1.85	0.78	Not included in analysis		
**Histological type**						
AD vs. non-AD	4.45	1.35–14.6	0.014	4.91	1.43–16.7	0.011
**p-TNM stage**						
Stage IIIA vs. Stage II	7.79	1.85–32.6	0.005	5.48	1.29–23.3	0.021
**Chemotherapeutic regimen**						
CDDP- vs. CBDCA-based	0.62	0.30–1.25	0.18	Not included in analysis		
**Number of treatment cycle**						
3,4 vs. 1,2	0.61	0.25–1.50	0.28	Not included in analysis		

Analyses were performed using Cox proportional hazard regression.

CI = confidence interval; HR = hazard ratio.

See Tables 1 and 2 legends for expansion of abbreviations.

## Discussion

The aim of AC in patients with resected NSCLCs is to eradicate micrometastasis and improve their survival. In the present study, we focused on the patients with completely resected stage II and IIIA NSCLC treated with AC, and estimated the prognostic impact of nestin expression on survival. We have demonstrated that nestin expression appears to be associated with poorer prognosis and is an independent prognostic factor for survival in patients receiving AC. In addition, we evaluated the prognostic impact of nestin on survival in 59 cases (8 nestin-positive cases) with AC-naïve, completely resected stage II and IIIA NSCLCs in populations of our previous study [7] and nestin expression was not found to be significantly associated with poor prognosis (*P* = 0.724) in AC-naïve cohort. It may further emphasize that nestin is a marker for chemoresistance. Although nestin expression was not a significant predictive factor for DFS, nestin-positive cases appear to have recurred earlier than nestin-negative cases in the relative early phase after surgery (for instance at 24 months). According to these results, nestin expression may influence chemoresistance in patients with recurrent NSCLC after surgery. In the treatment of patients with postoperative recurrence, patients with EGFR mutations have a significantly longer survival than those with wild type EGFR when treated with EGFR-TKIs [16]. In the present study, the status of EGFR mutations did not different between nestin-positive and nestin-negative groups. Moreover, there was no difference in OS between nestin- positive and nestin-negative groups. Thus, in the present study, nestin expression in patients with EGFR mutations did not influence survival.

To investigate the relationship between nestin expression and chemoresistance, we further examined ABCG2- and EMT-related marker expression. ABCG2, also known as breast cancer resistant protein, is a transporter protein and plays a major role in chemoresistance [17]. Lechner et al. reported that co-expression of nestin and ABCG2 was observed in pluripotent side population cells from adult human pancreatic islets [18]. However, in the present study, nestin expression was not associated with ABCG2 expression.

Previous studies reported that EMT play a role in cancer progression and drug resistance [19]. The hallmarks of EMT *in vitro* and *in vivo* include the acquisition of a spindle-like/fibroblastic morphology, the gain of mesenchymal markers (vimentin, N-cadherin, α-smooth muscle actin), and the loss of epithelial cell surface markers and cytoskeleton components (E-cadherin, ZO-1, claudins, occludins, cytokeratins) [20]. Zhao et al. reported that nestin regulates the EMT process in breast cancer [21]. Therefore, we analyzed E-cadherin and vimentin expression to investigate the relationship between nestin expression and EMT, and demonstrated that nestin expression is significantly associated with loss of E-cadherin expression and gain of vimentin expression, and is significantly associated with the complete type in the EMT phenotype according to expression of E-cadherin and vimentin. PC is defined as poorly differentiated NSCLC containing spindle cells and/or giant cells, and the pleomorphic component should comprise at least 10% of the neoplasm [9]. Mochizuki et al. proposed that EMT is involved in the pathogenesis of PC [22]. In surgical samples of this study, nestin expression in tumor cells was observed in 3 of 3 (100%) PCs. Several studies reported that nestin is a putative marker of the cancer stem cell (CSC) phenotype [6]. In the relationship between CSC and EMT, CSC as well as cells undergoing EMT are considered to be more resistant to toxic injuries and chemoradiation therapy than differentiated daughter cells, and EMT may be involved in the development of CSC and the characteristics of chemoresistance [19]. Several lines of evidence raise the possibility that nestin expression is linked to EMT in the mechanisms of chemoresistance. Further study is needed to clarify the molecular mechanisms by which nestin regulates the EMT process in NSCLC.

There are a few limitations to our study. First, the sample size of our study is relatively small, and the follow-up duration is relatively short. Therefore, a larger population and longer follow-up duration will be needed in future to clarify the findings of the present study. Second, 32.3% of patients were treated with a carboplatin (CBDCA)-based regimen in the present study. The Lung Adjuvant Cisplatin Evaluation meta-analysis demonstrated that adjuvant cisplatin (CDDP)-based chemotherapy is of benefit in completely resected NSCLC [23]. However, Williams et al. reported that a CBDCA-based regimen remained the most frequently used platinum agent among patients treated with AC in 2001 through 2005 [24]. Similarly, CBDCA-based regimen was frequently selected in our hospital before 2006. Moreover, in practice, some patients have significant comorbid disease burden and are not able to tolerate cisplatin. Although substituting CDDP with CBDCA is controversial, some studies reported that CBDCA-based and CDDP-based regimens have similar survival outcomes in patients treated with AC [24, 25]. Indeed, the present study showed that the chemotherapeutic regimen (CBDCA-based or CDDP-based regimen) is not a prognostic factor for OS in univariable analysis.

## Conclusions

We revealed that nestin expression is significantly associated with EMT-related markers such as loss of E-cadherin and gain of vimentin, and is a prognostic indicator of a poorer survival probability in NSCLC patients receiving AC, although its prognostic significance still requires confirmation with larger patient populations. Further understanding of the links between nestin, EMT, and chemoresistance may lead to the identification of novel therapeutic targets for the prevention of resistant to conventional chemotherapy in NSCLC.

## Abbreviations

ABCG2: ATP-binding cassette sub-family G member 2
AC: adjuvant platinum-based chemotherapy
AD: adenocarcinoma
CBDCA: carboplatin
CDDP: cisplatin
CSC: cancer stem cell
EMT: epithelial to mesenchymal transition
NSCLC: non-small cell lung cancer
PC: pleomorphic carcinoma
p-TNM: pathologic TNM
SCC: squamous cell carcinoma
